# Regulating Divergent Transcriptomes through mRNA Splicing and Its Modulation Using Various Small Compounds

**DOI:** 10.3390/ijms21062026

**Published:** 2020-03-16

**Authors:** Ken-ichi Fujita, Takaki Ishizuka, Mizuki Mitsukawa, Masashi Kurata, Seiji Masuda

**Affiliations:** Division of Integrated Life Sciences, Graduate School of Biostudies, Kyoto University, Kyoto 606-8502, Japan; kfujita.sincere1011@gmail.com (K.-i.F.); clarityaxel@gmail.com (T.I.); mitsukawa.mizuki.55m@st.kyoto-u.ac.jp (M.M.); masashikurata@gmail.com (M.K.)

**Keywords:** alternative mRNA splicing, cancer, neurodegenerative disorder, splicing inhibitor, food-derived compound

## Abstract

Human transcriptomes are more divergent than genes and contribute to the sophistication of life. This divergence is derived from various isoforms arising from alternative splicing. In addition, alternative splicing regulated by spliceosomal factors and RNA structures, such as the RNA G-quadruplex, is important not only for isoform diversity but also for regulating gene expression. Therefore, abnormal splicing leads to serious diseases such as cancer and neurodegenerative disorders. In the first part of this review, we describe the regulation of divergent transcriptomes using alternative mRNA splicing. In the second part, we present the relationship between the disruption of splicing and diseases. Recently, various compounds with splicing inhibitor activity were established. These splicing inhibitors are recognized as a biological tool to investigate the molecular mechanism of splicing and as a potential therapeutic agent for cancer treatment. Food-derived compounds with similar functions were found and are expected to exhibit anticancer effects. In the final part, we describe the compounds that modulate the messenger RNA (mRNA) splicing process and their availability for basic research and future clinical potential.

## 1. Introduction

In eukaryotic cells, protein-coding genes are transcribed as pre-messenger RNA (mRNA) in the nucleus and pre-mRNA undergoes several RNA processing steps, such as 5′-capping, splicing, and 3′-end processing. These gene expression processes are tightly coordinated with each other to achieve efficient and accurate gene expression [1]. After the mRNA processing steps, the mature mRNA is exported from the nucleus to the cytoplasm for translation. Human transcriptomes are more divergent than genes. This divergence is derived from various isoforms arising from alternative splicing, which is an essential biological process for considerable proteomic diversity and complexity despite the relatively limited number of human genes [2]. Furthermore, alternative splicing has important roles not only for expressing proteins through transcript diversity but also for regulating gene expression. Transcripts from most human protein-coding genes undergo one or more forms of alternative splicing. Alternatively spliced isoforms vary greatly from tissue to tissue [3]. Recent comprehensive analysis suggested that more than 40% of genes express multiple isoforms in a single tissue [4]. In particular, many isoforms containing alternative exons are expressed in neurons; thus, the brain displays the most complex pattern of alternative splicing [5]. Alternative splicing contributes to cell differentiation and lineage determination, tissue identity acquisition and maintenance, and organ development [6,7,8]. Thus, alternative splicing is considered to be a key mechanism for regulating gene expression networks, as well as for human diversity or sophistication. 

**Figure 1:**
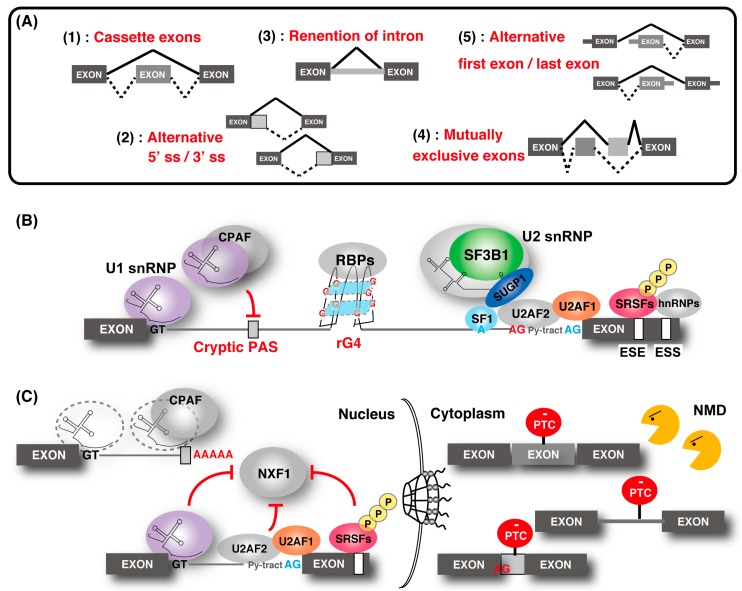
Regulation of gene expression through alternative splicing. (**A**) Five types of alternative splicing events. "Cassette exons" means inclusion or skipping of an exon. (**B**) Outline of the alternative splicing control by various factors. The U1 ribonucleoprotein (snRNP) function to recognize the 5′ splice site (ss) and the cryptic polyadenylation signals (PASs) with cleavage and polyadenylation factors (CPAFs). Splicing factor 1 (SF1), U2 snRNP auxiliary factor 1 (U2AF1), and U2AF2 bind the branch point (BP; shown as a blue “A”), polypyrimidine tract (shown as “Py-tract”), and 3′ ss (shown as a blue “AG”), respectively. U2 snRNP containing splicing factor 3B1 (SF3B1), SURP and G-patch domain containing 1 (SUGP1) displaces SF1 and binds to the BP. SR protein families and hnRNPs recognize the exonic-splicing enhancer (ESE) and exonic-splicing silencer(ESS) elements, respectively, and contribute to the recognition of the splice site and the spliceosome assembly by splicing factors. The cryptic 3′ ss (shown as a red “AG”) recognition is repressed by hnRNP. RNA G-quadruplex (rG4) structures affect splicing by acting as RNA-binding protein motifs. (**C**) Association of alternative splicing and gene regulation. Without U1 snRNP, the CPAF recognizes intronic PASs and generates short transcripts due to premature cleavage and polyadenylation. Some intron-containing transcripts are associated with U1 snRNP, U2AF2, and SR proteins, and they are tethered in the nucleus. Intron-containing transcripts, which are exported to the cytoplasm, often contain a premature termination codon (PTC) and are eliminated by nonsense-mediated messenger RNA (mRNA) decay (NMD). Similarly, other alternative and cryptic-spliced transcripts with PTC are also degraded by NMD.

The spliceosome, a multimegadalton ribonucleoprotein (RNP) complex comprising five snRNPs and numerous proteins, carries out splicing of pre-mRNA molecules to remove introns, then conjoins exons in different arrangements that potentially encode alternative protein isoforms [9]. Spliceosome complexes are assembled at the splice sites in a pre-mRNA transcript, involving a stepwise assembly pathway of the U1, U2, and U4/5/6 snRNP spliceosome subunits. The U1 snRNP binds the 5′ splice site (5′ ss), and splicing factor 1 (SF1) and U2 snRNP auxiliary factor (U2AF) 1/2 bind the branch point (BP), polypyrimidine tract, and the 3′ ss, respectively (Figure 1). After binding, the U2 snRNP containing the splicing factor 3A (SF3A) and splicing factor 3B (SF3B) subcomplex stably associates with BP, following the engagement of U4/U6 and U5 snRNPs in the form of a tri-snRNP particle. This leads to the destabilization of the U1 and U4 snRNPs. These conformational and compositional rearrangements of spliceosomal components result in the activated spliceosome; this emergence triggers two sequential transesterification reactions to produce the spliced-mRNA. Additional interactions that contribute to the recognition of intron–exon boundaries and/or the spliceosome assembly are mediated by elements of the cis-acting exonic-splicing enhancer (ESE) and intronic-splicing enhancer, and exonic-splicing silencer (ESS) and intronic-splicing silencer, which are recognized by auxiliary splicing factors, including the Ser/Arg-rich (SR) proteins (hereafter described as SRSFs) and heterogeneous nuclear ribonucleoproteins (hnRNPs) [10]. Strict recognition of the splicing site by these factors enables individual alternative splicing.

Splicing contributes to precise gene regulation in connection with other forms of processing in the nucleus. For example, splicing of first introns feeds back to transcription elongation and the efficiency of last intron removal affects cleavage and polyadenylation of mRNAs [11,12,13]. Coupling mRNA splicing to mRNA export ensures efficient nuclear export of mature mRNPs for translation in the cytoplasm mediated by the evolutionarily conserved transcription and export (TREX) complex [1,14]. The TREX complex is recruited to mRNA in a splicing-dependent manner via splicing factor and UAP56, and it triggers the association of nuclear RNA export factor 1 (NXF1), which is a final mRNA export factor, onto the export competent mRNA. Recently, a molecular mechanism that suppresses the recruitment of NXF1 to incompletely spliced mRNAs was partly demonstrated [15,16,17]. In addition, gene regulation through nonsense-mediated mRNA decay (NMD) was shown [18,19,20,21,22,23,24].The aberrant transcript with the pre-mature termination codon, derived from abnormal splicing, is removed by NMD. 

Mis-regulation of complicated alternative splicing is associated with cancers, and abnormal expression or mutations in splicing factors contribute to tumorigenesis and neurodegenerative disorders [25]. Recently, various compounds with splicing inhibitor activity were established. These chemical compounds are expected to act not only as a biological tool to investigate complicated splicing processes but also for anti-cancer drugs targeted to the splicing machinery. Food-derived compounds having similar functions were also identified and are receiving attention. In this review, we summarize these compounds and discuss their potential validity in physiological function.

## 2. Understanding the Diversity of Transcriptomes by Controlling mRNA Splicing

Alternative splicing is typically classified into five types (Figure 1A): (1) inclusion or skipping of individual “cassette” exons, (2) switching between alternative the 5′ and 3′ ss, (3) differential retention of introns (RI), (4) mutually exclusive splicing of adjacent exons, and (5) alternative splicing coupled with alternative first or last exons [3,26].

Alteration of alternatively spliced isoforms often results in changes in translational efficiency and protein isoforms that exhibit a different enzymatic activity, location, and protein–protein interaction. Thus, alternative splicing contributes to the diversity of protein function [27,28]. In particular, the intrinsically disordered region (IDR), also referred to as the low-complexity region, produced by alterative splicing, attracted attention in recent studies [29,30]. The IDR is characteristic for protein–protein interaction and regulated by post-translational modifications. The role of alternative splicing in diversifying protein interaction capabilities was reported because region encoding of the IDR is often controlled by alternative splicing [31,32,33]. The IDR, which contains the abundant GY motif, was implicated in the formation of higher-order protein complexes that can undergo phase separation and assemble into membraneless organelles and fibrillar-like structures [29,34,35]. Alternative splicing events within corresponding mRNA that encode these IDR regions are significantly enriched in members of the hnRNP A and D families, which have diverse roles in splicing and other RNA biological processes. These alternative splicing events arose in mammals through evolution, and they are expected to play an important role in controlling splice site recognition by the hnRNP A and D families [36].

### 2.1. Relationship between Alternative SPLICING and NMD and mRNA Localization

Alternatively spliced isoforms enable distinct regulatory properties in the cell, such as individual cell mRNA stability and localization (Figure 1B,C) [37]. Regarding cell mRNA stability, the mechanism for eliminating mRNA, which alters the reading frame, is NMD. NMD is an evolutionarily conserved cellular quality control mechanism that inspects a premature termination codon (PTC) introduced by the change in the reading frames on the mRNA. After recognizing the PTCs, mRNA containing PTC is cleaved and eliminated from the transcriptome. PTCs are particularly problematic because they often result in the production of nonfunctional and/or dominant-negative proteins [18,19]. 

Living organisms also regulate gene expression by efficiently using the NMD mechanism. For example, SRSF3 is a member of the SR protein family that strictly regulates its own expression by controlling the inclusion of PTC-containing cassette exons in its own transcript using alternative splicing. This is referred to as a “poison cassette exon” because it leads to transcript degradation by NMD [20]. Not only SRSF3 but all species in the SR protein families control the regulation of their own expression through ultraconserved poison cassette exons [21]. These features are also observed in some RNA-binding proteins (RBPs), including the hnRNP family [22,23,24].

The RI attracted attention in recent years because it was demonstrated that introns contribute to the regulation of gene expression, nuclear mRNA export, and the production of new isoforms [38]. RI products can be roughly classified into two types. One class is exported to the cytoplasm without retention in the nucleus. This class often contains PTCs, which are frequently observed in genes encoding splicing factors and RBPs and generally serve to downregulate protein expression by irreversibly eliminating the PTC-containing mRNAs [39,40,41]. In some cases, these transcripts without PTC undergo a translation process. These were identified by ribosomal-profiling analysis and characterized by short introns in the 5′ UTR region, as well as enrichment of genes involved in the cell cycle [42]. The other class is characterized as an intron-containing transcript stably retained in the nucleus and was distinctly defined as the detained intron (DI) by Boutz and colleagues [41]. The DI products are insensitive to NMD, and they negatively affect protein expression by preventing the respective mRNAs from being translated because they are retained in the nucleus. Surprisingly, various stimuli, such as DNA damage or neuronal activation, trigger the rapid post-transcriptional splicing of the DI. As a result, the spliced DI product is immediately exported and undergoes translation [41,43,44,45,46]. In neuronal cells, genes associated with neuronal activation tend to be long, and their transcriptional regulation is insufficient for acute phase expression because it takes time to make a full-length transcript. Thus, divergent transcripts with the DI synthetized from corresponding genes are pooled in the nucleus beforehand. They are spliced and exported to the cytoplasm in response to a stimulus [46]. These findings suggest that the DI has an important role in acute phase gene expression.

Incompletely spliced mRNAs are retained within nuclear speckles (NS) in mammalian cells [17]. NS are membraneless nuclear domains enriched in mRNA splicing factors, 3′ processing factors, and export factors, and they are located in the interchromatin regions of the nucleoplasm. Recent studies suggested that NS act as a hub to coordinate all nuclear mRNA processing steps and quality control steps [47]. How mRNPs gain export capacity and how they remain in the nucleus are current areas of active research. The two mechanisms for nuclear retention are expected to be (1) active anchoring within the nucleus and (2) prevention of export factors being recruited. 

Early spliceosomal components, U1 snRNP and U2AF2 and SR proteins, are reported to be associated with nuclear retention [17]. In one study, depletion of U1 snRNP protein component, U1-70K, and U2AF2 prevented nuclear retention of unspliced human β-globin reporter transcripts and caused their leakage into the cytoplasm [48]. Tethering of U1-70K and U2AF2 reporter transcripts also caused nuclear retention via their RS domain, which is rich in arginine and serine repeats. In addition, depletion of U2 snRNA or specific subunits of the SF3B complex did not cause this prevention, indicating that binding of the U2 snRNP is not required for nuclear retention. Moreover, it was shown that unspliced polyadenylated RNAs that accumulate within NS were still associated with stalled inactive spliceosomes [49]. SR proteins generally have an RS domain that can be phosphorylated at multiple positions. The phosphorylation status of the SR protein is regulated by SR protein kinase and cdc2-like kinase families, and protein phosphatase 1 contributes to nuclear retention and splicing regulation activities [15,16,50]. Phosphorylated SR proteins are associated with splicing sites to facilitate splicing and are dephosphorylated during splicing. After completion of splicing, SR proteins are again phosphorylated to be recycled for the next splicing. Some SR proteins, in addition to the TREX complex, support mRNA export only in their dephosphorylated state as a result of productive splicing [15,16,17]. Therefore, in the case of an incomplete splicing condition, phosphorylated SR proteins associated with the splicing site act as retention factors that do not recruit NXF1 for export (Figure 1C). These spliceosomal components commonly contain IDR regions with the RS domain, which suggests that the regulation of protein–protein interaction through the IDR region plays an important role in mRNA retention in NS. Some intron-containing transcripts are efficiently exported to the cytoplasm because they directly recruit NXF1 and override nuclear retention [51]. Consistently, a subset of intron-containing cellular transcripts bound by NXF1 and SR proteins are stably detectable in the cytoplasm [52]. These findings suggest that some of the intron-containing transcripts are efficiently exported by recruiting NXF1 and SR proteins and by escaping from retention in the nucleus. 

### 2.2. Cryptic Splicing

The eukaryotic genome has a large number of cryptic splice sites that are rarely used under the normal conditions but can be potentially recognized. They are often flanked by a high density of corresponding motifs that bind to hnRNPs and some RBPs to repress their splicing recognition [30,53]. For example, hnRNP C can suppress cryptic exon recognition by binding to U-tracts in Alu elements within the intron [54]. NOVA alternative splicing regulator (Nova), a neuron-specific splicing factor, represses splicing by binding to long clusters of YCAY motifs [55]. TAR DNA-binging protein 43 (TDP-43) and polypyrimidine tract-binding protein (PTBP)1/2, which are RBPs with various molecular functions related to RNA metabolism, and hnRNP L act as repressors by binding to specific repeats [56,57,58]. RBM17 is implicated in cancer and a neurodegenerative disease, and it can repress cryptic splice site recognition [59]. The recognition sequence of RBM17 overlaps with the target sequence of the cryptic exon suppressed by TDP-43. U2 snRNP is also believed to be involved in the repression of cryptic splice site recognition [60,61,62,63,64]. These potential but inactive splice sites can be recognized when an authentic strong splice site is mutated or when there are defects in the hnRNPs and some RBPs [59]. The breakdown of the retention mechanism is linked to cancer and neuronal diseases. We discuss these linkages in the next section.

### 2.3. Telescripting by U1-CPAFs

To prevent abnormal mRNA synthesis by mis-splicing, “telescripting” was identified, which is accomplished by the unique role of U1 snRNPs in the central regulation of splicing and 3′ end cleavage/polyadenylation. It was reported that inhibition of the U1 snRNP caused not only splicing inhibition but also premature cleavage and polyadenylation in numerous pre-mRNAs at cryptic polyadenylation signals (PASs), frequently found in introns adjacent to the transcription start site (less than 0.5 kilobases) [65,66]. This event was not observed in U2 snRNP inhibition, and it was revealed that the U1 snRNP combined with intronic PASs form the complex of the U1 snRNP with cleavage and polyadenylation factors (U1-CPAFs), which are distinct from U1-spliceosomal complexes because of the lack of essential splicing factors [67]. U1-CPAFs co-transcriptionally protect pre-mRNAs from premature cleavage and polyadenylation (PCPA) at cryptic PASs in introns, thus ensuring transcriptome integrity (Figure 1C). This function is termed “telescripting” and is separate from the role in splicing [65,66]. In addition, U1 telescripting determines mRNA length and confers transcriptional directionality from bidirectional promoters [66,68,69,70]. The U1 snRNP is abundantly expressed compared with other snRNPs in human. These findings reveal a critical splicing-independent function of U1 snRNP.

### 2.4. Splicing Regulation by RNA G-Quadruplex

RNA secondary structures were shown to play key roles in gene expression through regulating various forms of mRNA metabolism [71,72]. Regarding the regulation of mRNA metabolism, one stable nucleic acid structure is the G-quadruplex, which is formed within guanine-rich sequences. This unique structure can be formed within a single strand or between multiple strands of RNA or DNA, where four G-tracts of two or more guanines, separated by short stretches of other nucleotides, are assembled in layered loops bound together through Hoogsteen hydrogen bonding. G-quadruplexes were initially thought to focus on DNA, but they were recently found on RNA molecules as well [73]. Initially, several studies demonstrated that the RNA G-quadruplex (rG4) structures in 5′ UTRs act as regulatory elements for translation [74,75,76]. rG4 in 5′UTRs of mRNAs, such as NRAS, KRAS, TRF2, FGF2, and VEGF can impair both cap-dependent and cap-independent translation [76,77,78,79,80]. Recently, it was reported that the regulation of rG4 folding by the cytoplasmic RNA helicase DHX36 was associated with translational efficiency [73,81].

rG4 was also reported to be significant in regulating nuclear mRNA processing, such as 3′ end processing [82,83,84], mRNA localization [85], and alternative splicing. Indeed, several studies provided experimental evidence that rG4 structures forming sequences proximal to the splice sites in introns affect the splicing and expression patterns of Bcl-xL, FMR1, and TP53 in human [86,87,88]. In addition to known linear RNA-binding motifs, rG4 was found to serve as an RNA-binding protein motif to mediate RNA processing. To elucidate the splicing control by rG4, research on the identification of factors recognizing rG4 is also been progressing. For example, the rG4 structure sequesters in hnRNP H, resulting in the local depletion of hnRNP H and, thus, disruption of hnRNP H-dependent splicing events occurs [89]. In addition, it was reported that alternative splicing regulation via the rG4 structure may control cellular processes that are important for tumor progression. hnRNP F regulates the CD44 isoform switch in a rG4-dependent manner, which is associated with an epithelial–mesenchymal transition and plays integral roles in normal development and cancer metastasis [90,91]. Profiling of rG4 revealed widespread and evolutionarily conserved rG4 structures in the human transcriptome [92]. The relationship between the splicing factor recognition of rG4 and regulation of alternative splicing is required for detailed research to elucidate the exact role of rG4 in alternative splicing.

## 3. Diseases Associated with Aberrant mRNA Splicing

Aberrant mRNA processing is an important causative factor in various diseases. Aberrant mRNA splicing underlies a growing number of human diseases, including inherited disorders, cancer, diabetes, and neurodegenerative diseases [10]. Aberrant RNA splicing is caused by mutation of the trans-acting mRNA splicing factor and the cis-element, which is an essential sequence for the binding of splicing regulatory proteins and trans-acting mRNA splicing factor. Previous studies revealed that a relationship between genetic diseases with abnormal splicing is associated with mutations in cis-element and trans-acting factors [25]. Abnormalities in core constituents of spliceosome formation also underlie a discrete set of diseases, including neurodegenerative disorders and cancer. In this section, we discuss the interaction between mRNA splicing factor mutations and disease. 

### 3.1. Mutation of Spliceosomal Components and Cancer

Cancer has several typical characteristics, such as abnormal proliferation and alterations in cellular metabolism. The acquisition of these features is driven by changes in gene expression [93]. It was reported that gene regulation disorder caused by mRNA splicing factor mutation is linked to the progression of various cancers. 

**Figure 2:**
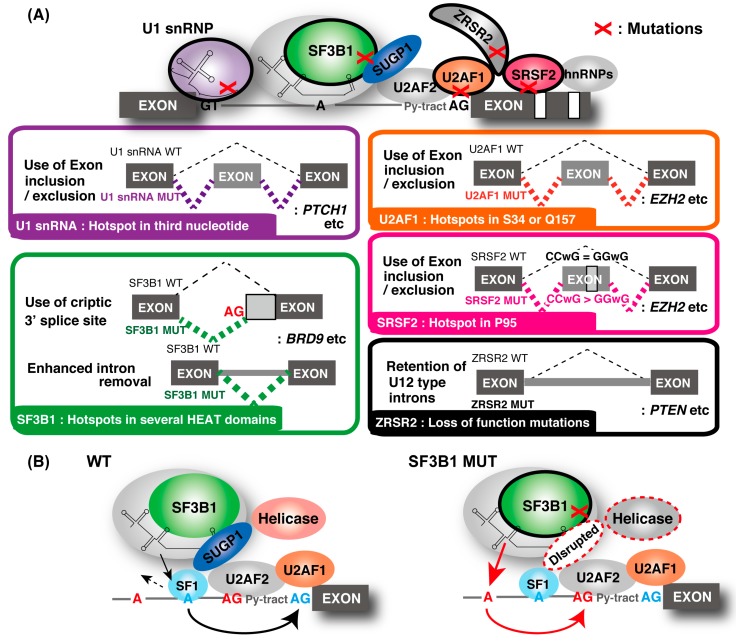
Mutations in splicing factors and their impact on splicing. (**A**) Alteration of splicing caused by splicing factor mutations are shown in the boxes. U1 snRNP: purple, SF3B1: green, U2AF1: orange, Ser/Arg-rich (SR) protein 2 (SRSF2): pink, zinc finger CCCH-type, RNA-binding motif and serine/arginine-rich 2 (ZRSR2): black. Mutations in U1 snRNA cause an alteration in the splicing pattern from the canonical 5′ ss to a slightly different 5′ ss. SF3B1 mutation induces cryptic 3′ ss usage (shown as a red “AG”) and enhances intron removal. U2AF1 mutations frequently alter the usage of cassette exons. SRSF2 mutations enhance the greater binding affinity to CCwG than to GGwG in ESE, which are equally recognized by wild-type SRSF2. ZRSR2 mutations induce aberrant retention of U12-type introns. (**B**) Recognition of the cryptic 3′ ss induced by the mutation of SF3B1. Under normal conditions, U2 snRNP containing wild-type (WT) SF3B1 associates with SUGP1, displaces SF1 by activating RNA helicases and uses a canonical BP and 3′ ss (shown as a blue “A” and “AG”) for splicing. By contrast, U2 snRNP containing SF3B1 mutants disrupt the association with SUGP1, resulting in the use of upstream BP and cryptic 3′ ss (shown as a red “A” and “AG”) for splicing.

Frequent and recurrent mutations are found within the early components of the RNA splicing machinery. For example, mutations in *SF3B1*, *U2AF1*, *SRSF2*, and *zinc finger CCCH-type, RNA-binding motif and serine/arginine-rich 2 (ZRSR2)* were found in a variety of hematological malignancies, including myelodysplastic syndromes (MDSs) and chronic lymphocytic leukemia (CLL), and they are mutually exclusive [94]. Heterozygous hotspot missense mutation was common characteristic for *SF3B1*, *SRSF2*, and *U2AF1* (Figure 2A). Mutations in *ZRSR2* throughout the coding sequence caused loss-of-function mutations [95]. Similar mutations were reported with a lower frequency in solid tumors [96]. Recently, recurrent hotspot mutations at the third nucleotide of *U1 snRNA* were found in several cancer types, including in medulloblastoma, with high frequency [97]. Further investigation revealed that hotspot U1 mutations were present in about 50% of sonic hedgehog (SHH) medulloblastomas, which represents one group of medulloblastomas [98]. In addition, mutations were not present across other subgroups of medulloblastoma, indicating that U1 snRNA mutations are highly recurrent in and extremely specific to SHH medulloblastoma. It was reported that 119 splicing factor genes carry putative driver mutations in one or more cancer types from tumor cohort studies [99]. These reports suggested that spliceosomal mutations were considered a new hallmark and driver of tumorigenesis rather than merely passenger mutations [1]. Mutations of various mRNA splicing factors were globally analyzed and shown to affect gene expression. In addition, cancer-specific splicing changes are increasingly recognized as contributing to tumorigenesis via various mechanisms.

#### 3.1.1. SF3B1

SF3B1 is a member of the SF3B complex within the U2 snRNP and plays a pivotal role in the early stages of spliceosome assembly and BP recognition [100]. Hotspot mutations in SF3B1’s HEAT domains were reported in many tumor types. These mutations induced the cryptic 3′ ss usage currently recognized as the most frequent splicing alteration [60]. These SF3B1 mutants are called change-of-function mutants because SF3B1 knockdown or overexpression does not reproduce these forms of aberrant splicing [61]. Nearly half of the aberrant mRNA transcripts are degraded by NMD, resulting in the downregulation of gene expression [60]. There are several reports on the splicing control mechanism by SF3B1 mutants. Mutant SF3B1 preferentially recognizes alternative BPs upstream of the canonical BP(s), which results in deregulated usage of an alternative 3′ ss being weakly dependent on U2AF1 [61]. Because SF3B1 mutation did not alter the SF3B1–U2AF complex formation and affinity with RNA [101], U2AF1 hotspot mutations described later did not lead to the same aberrant splicing phenotype, indicating that cryptic 3′ ss usage was specifically induced by SF3B1 mutants.

Structural analysis of the SF3B1 complex revealed that SF3B1’s HEAT domain was important for multiple contacts with the BP-binding proteins [101]. However, it was unknown how SF3B1 mutations affect the protein interactions in the spliceosome because hotspot mutations did not affect the stability of the SF3B1–U2AF complex and the affinity with RNA. Recently, it was reported that hotspot mutations in SF3B1 specifically disrupted the interaction with the spliceosomal protein, SURP and the G-patch domain-containing 1 (SUGP1), without the interference of other SF3B1-associated proteins [64]. SUGP1, previously known as splicing factor 4 [102], has two tandem SURP domains and a G-patch domain. SURP domains interact with SF1, and G-patch domains were shown to activate RNA helicases for ATP hydrolysis. Both domains are required for BP recognition by the SF3B1-containing U2 snRNP [103,104,105]. These findings strongly suggest that SUGP1 is involved in the BP recognition process. In fact, knockdown of SUGP1, but not any other members of SF3B1-associated proteins, recapitulated the aberrant splicing induced by mutant SF3B1, indicating that SUGP1 acts as a splicing regulator contributing to aberrant splicing. Those studies suggested the model shown in Figure 2B for SUGP1 function. SUGP1 is important for accurate BP recognition by the U2 snRNP with SF3B1, SF1, and U2AF. SUGP1 associates with and activates an unknown RNA helicase required for the displacement of SF1 [103], allowing base pairing between the canonical BP and U2 snRNP. Furthermore, SUGP1 overexpression partially rescued the splicing abnormalities induced by mutant SF3B1. Several mechanisms of abnormal splicing by SF3B1 were proposed [60,61,106], implying that the structural changes of SF3B1 induced by mutations are substantial and need to be solved. Those reports suggested that the understanding of how SUGP1 restores the assembly of the mutant spliceosome can be used to develop a potential cure for mutant SF3B1-driven cancers.

In addition to cryptic 3′ ss usage, widespread reduction of intron-retaining isoforms was also frequently identified in mutated SF3B1 samples [63]. The mutant SF3B1-associated decrease in intron retention was not due to the activated degradation of intron-retaining transcripts by NMD but was caused by the enhanced splicing of retained introns. The effect of this abnormality on cells is not yet known. To understand the relationship between SF3B1 abnormalities and tumorigenesis, studies on the pleiotropic splicing abnormalities are necessary.

**Figure 3:**
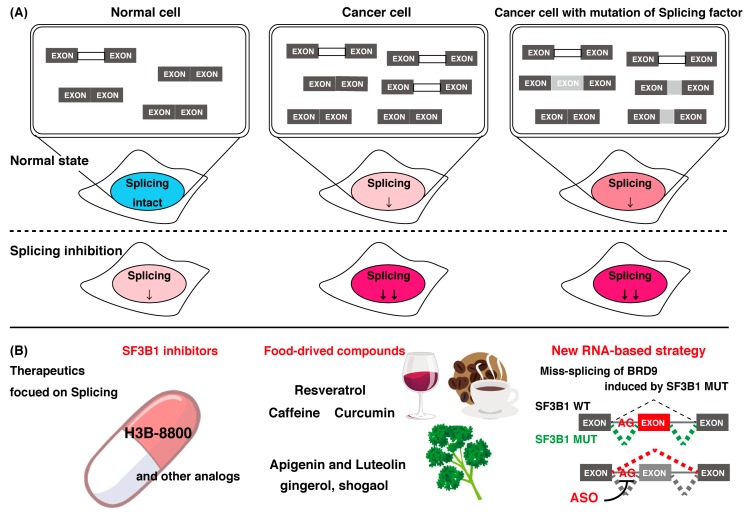
Availability of compounds with splicing inhibition activities. (**A**) Effects of splicing inhibitors on normal cells, cancer cells, and cancer cells with mutations in splicing factors. Cancer cells have more transcripts than normal cells, resulting in splicing stress. In addition, cells with mutations in the splicing factor cause splicing abnormalities. These splicing factor mutations are heterozygous, suggesting that wild-type splicing factors have an essential role in cell survival in these cancers. Cancer cells with or without splicing factor mutations are, thus, more sensitive to splicing inhibition than normal cells. (**B**) The anti-cancer effect of various splicing inhibitions. SF3B1 inhibitors, such as H3B-8800, are anticipated for clinical use. Food-derived compounds with similar splicing inhibition activity were also reported to have anti-cancer effects, and they are expected to have cancer-preventing effects. In addition, inhibition of mis-splicing on BRG9, which is widely observed in cancers with an SF3B1 mutation, using antisense oligonucleotides (ASO) is expected to be a new strategy for cancer treatment.

Among these types of abnormal splicing regulation, it was reported that diverse SF3B1 mutations converge on the repression of BRD9, which is a core component of the recently described noncanonical BAF chromatin-remodeling complex, which plays a suppressor role in tumorigenesis [62]. That study found that mutant SF3B1 recognizes an aberrant intronic BP within BRD9, thereby inducing the inclusion of a poison exon. This results in the repression of BRD9 mediated by NMD, thus promoting tumor growth and metastasis. These results indicate that the poison exon of BRD9 becomes a target of therapeutic potential in SF3B1-mutated cancers. Indeed, tumor-suppressive effects of correcting BRD9 mis-splicing with multiple methods, including antisense oligonucleotides, were achieved [62]. In general, it is thought that the multiple splicing alterations may cooperatively contribute to the pathogenesis of cancers. Thus, recent studies suggest the potential of new therapeutics targeting mis-spliced transcripts in anticancer treatment (Figure 3B).

#### 3.1.2. SRSF2

SRSF2 is a member of the SR protein family that promotes exon inclusion by binding to the ESE sequences, CCNG or GGNG, through its RNA recognition motif (RRM) domain [9]. SRSF2 mutations consistently affect the P95 residue and have increased binding affinity toward CCNG, but have decreased binding affinity toward the GGNG sites [107,108,109]. As a result, global alterations of splicing, including exon inclusion and exclusion, are induced by SRSF2 mutation [63,107,108,109]. SRSF2 mutants promote the splicing alteration of key hematopoietic regulators, such as enhancer of zeste homolog 2 (EZH2), which impairs hematopoietic differentiation. Because SRSF2 mutation did not affect protein–protein interactions with key splicing factors in one study [107], it was assumed that SRSF2 mutations affect the conformation of the RNA-binding domain [108,110]. The structural information of SRSF2 will uncover the mechanism of abnormal splicing induced by SRSF2 mutation.

In addition to the splicing alteration in SRSF2 mutant cells, DNA damage is also induced [111]. SRSF2 mutation causes impairment of transcription pause release and induces R-loop formation. Accumulation of R-loops often results in increased cellular stress that leads to genomic instability [112]. The chromosomal instability could be a major driving force in tumorigenesis and cancer evolution.

#### 3.1.3. U2AF1

Recurrent hotspot mutations in *U2AF1* at the Zn-finger domain (S34 or Q157) were reported [113,114]. These mutations altered the RNA-binding specificity [114,115,116,117] and the splicing kinetics [118], resulting in a wide variety of splicing outcomes. Changes in the cassette exon were observed most frequently in these mutations [94,115,119,120,121,122,123]. Although it was reported that the U2AF1 mutant also induces abnormal splicing of EZH2 [63]; other splicing alterations are not linked to a phenotype, leading some investigators to propose a nuclear RNA that processes defects such as alterations in 3′UTRs [124] or an increase in R-loops induced by the U2AF1 mutant [111,125].

#### 3.1.4. U1 snRNP

A recurrent A-to-C and A-to-G mutation in the third base of the *U1 snRNA* occurs at part of a highly conserved 5′ ss recognition sequence (nucleotides 3–10) of U1, which forms base pairs directly with the 5′ ss [97,126]. This mutation changed the preferential A–U base-pairing between the U1 snRNA and the 5′ ss to C–G base-pairing, and it caused alternative 5′ cryptic splicing and alteration of the splicing pattern in multiple genes, including known drivers and repressors of cancer [97,98]. These events are thought to be specific to U1 snRNA mutations because SF3B1 mutations tend not to share these abnormal types of splicing [97].

As a novel link between cancer progression and splicing factors through noncanonical roles, the association with splicing factor U2AF1 and translational regulation was reported [127]. That study revealed that U2AF1 and U2AF2, bound to the 5′ UTR, were located on hundreds of mature RNA in the cytoplasm and functioned as a translational repressor. The recurrent cancer-associated hotspot mutation (S34F) in U2AF1 caused loss of binding and translational de-repression, resulting in increased synthesis of the IL8 chemokine, which contributes to metastasis, inflammation, and cancer progression [128,129]. In addition to the contribution of U2AF1, it was reported that SF3B4, a component of the SF3B complex, functions as a cofactor for p180, an essential factor for high-rate protein synthesis on the ER, and it plays a key role in enhanced translation [130]. These findings suggest that mRNA splicing factors have multiple roles in translation, and cancer-related mutations of mRNA splicing factors affect translation, as well as splicing.

### 3.2. Spliceosome Abnormality and Neurodegenerative Disorders

It is widely accepted concept that abnormalities of a number of RBPs are related to neurological diseases. Aberrant activity and localization of two RBPs, TDP-43 and FUS RNA binding protein (FUS), are implicated as the pathogenicity of amyotrophic lateral sclerosis (ALS) and frontotemporal dementia (FTD), two fatal neurodegenerative disorders that share clinical, genetic, and pathologic hallmarks [131]. These proteins were found to mediate a number of pathways related to RNA metabolism in the cytoplasm. In each disease, TDP-43 and FUS form mRNP aggregates in the cytoplasm [132,133,134]. The pathological consequences of mRNP aggregates are believed to be multifactorial in nature, but their roles are not yet completely defined. These aggregates disturb their normal functions by disrupting splicing [1]. 

Several reports demonstrate that spliceosomal components in the nucleus are mislocalized into an aggregate commonly observed in these neurodegenerative disorders [135,136,137]. The U1 snRNP, the most abundant FUS interacting complex, co-mislocalizes with FUS to the cytoplasm [135]. In addition, cytoplasmic aggregation of TDP-43 causes mislocalization of spliceosome components [136]. In zebrafish, knockdown of U1 snRNP components caused the truncation of the motor neuron axon, and similar phenotypes were observed by knockdown of FUS and survival motor neuron protein (SMN) [135]. Alzheimer’s disease (AD) is an age-related neurodegenerative disorder characterized by synaptic dysfunction, amyloid plaques, and neurofibrillary tangles formed by the aggregation of Tau protein encoded by the *microtubule associated protein tau (MAPT)* gene [138]. In the brains of patients with AD, increased aggregation of insoluble U1 snRNP was identified by proteomic analysis [137]. Previous studies showed that the U1 snRNP co-aggregates with Tau in the neurofibrillary tangles in human AD postmortem brain tissue [139,140]. Similar findings were reported using in vitro experiments and MAPT transgenic mice [141,142,143]. In *Drosophila*, panneuronal Tau expression triggers aggregation of U1-specific spliceosomal proteins [144]. That study suggested that mislocalization of spliceosomal components is also associated with these neurodegenerative disorders.

The functions of TDP-43 include repressing the splicing of nonconserved cryptic exons, maintaining intron integrity and preventing cell death [56,145]. RNA-seq analysis in human postmortem brain with TDP-43 mutations revealed cryptic exon expression [56]. This feature is common in ALS/FTD patients [56,138]. Whether the mislocalization of spliceosomal components induced by the aggregation of FUS or TDP-43 directly contributes to the cryptic splicing is still unknown. However, in AD, Tau pathology associates with splicing errors, including cryptic splicing and intron retention in human brains [144]. The mislocalization of the U1 snRNP-mediated aggregation of Tau causes a loss of nuclear U1 snRNP. A similar profile of cryptic splicing and intron retention was reported in Tau transgenic fly and small nuclear ribonucleoprotein-associated protein B (SmB), a component of snRNP, mutant fly [144,146]. Furthermore, mutation of SmB causes progressive neurodegeneration. The loss of the U2 snRNA also induced cryptic splice junctions and intron retention along with prominent cerebellar degeneration. These reports suggested that disruption or alteration of spliceosome function causes severe and toxic transcriptome formation and neurodegenerative diseases. In addition to the above evidence, findings showed that, preceding the onset of neurodegeneration, splicing changes were detectable in young flies [147]. Furthermore, direct manipulation of a core spliceosome component also caused neurodegeneration, which raises the possibility that splicing errors are likely a cause rather than a consequence of neurodegenerative disorders. The observation that alternative splicing occurred at the highest rate in the brain is consistent with the idea that the regulation of splicing in the brain has an important role in neuronal diversity and brain health [148]. Investigating connections between the unique role of core spliceosomal components and a molecular mechanism associated with neurodegenerative disorders will increase the understanding of therapeutic strategies targeting these factors.

## 4. Compounds that Control mRNA Splicing

As mentioned above, mRNA splicing is a complicated and dynamic process with a number of interacting splicing factors. One effective investigation strategy is to analyze the mechanism of mRNA processing using chemical compounds as biological tools that function to inhibit mRNA processes. In this section, we discuss the various compounds with splicing inhibitors discovered recently and modulators of rG4 formation.

### 4.1. Chemical Compounds That Control mRNA Splicing

Various compounds with inhibitory activity on splicing were identified from several microorganisms. Some of these compounds were found as cancer-specific effectors in early research, and the studies that followed revealed that the target proteins of these compounds were mRNA splicing factors. Synthetic derivatives of these compounds are, therefore, established as more effective splicing inhibitors. To date, the best-studied group of splicing inhibitors involves SF3B1 inhibitors, which contain pladienolide B (PlaB), spliceostatin A (SSA), GEX1A, and their analogues. We briefly introduce representative SF3B1 inhibitors and isoginkgetin, which inhibits a different target (Table 1).

#### 4.1.1. Representative SF3B1 Inhibitors and Isoginkgetin

SF3B1 inhibitors were individually identified as having distinct structures based on different assays. PlaB, isolated from a natural product derived from *Streptomyces platensis* Mer-11107, has cellular splicing inhibition activity [149]. E7107 has enhanced stability compared with PlaB and directly binds to SF3B3, a component of the SF3B complex. This results in the inhibition of SF3B1 and the impairment of U2 snRNP interaction with pre-mRNA [149]. Other studies reported that E7107 interacts with the SF3B complex in the branch point adenosine-binding pocket [150,151], which results in reducing the stability of early “A complex” formation by weakening the interaction between the U2 snRNA and pre-mRNA [152,153,154,155,156]. H3B-8800, an analogue of PlaB, was identified as a compound that competed with PlaB for binding to SF3B complexes. H3B-8800 received focus as a next-generation splicing inhibitor to enter clinical trials because of its similar, but not identical, activity to E7107, as described later [157].

SSA is a methylated derivative of the natural product, FR901464, which was isolated from the fermented broth of the bacterium *Pseudomonas* sp. as an anticancer compound [158]. In subsequent research, it was found that SSA binds to the SF3B complex, decreases the U2 snRNA interactions with the BP and results in the inhibition of splicing [153]. Sudemycin was designed based on the pharmacophore model between FR901464 and PlaB. Sudemycin shows splicing inhibition activity in a similar manner to SSA, and exhibits better chemical stability and half-maximal inhibitory concentration (IC_50_) values in cell lines [159,160].

GEX1A was originally isolated from a culture broth of *Streptomyces* sp. [161] and serves as a splicing inhibitor that specifically impairs the SF3B function by binding to SF3B1 [162]. Studies involved in the search for synthetic analogues of GEX1A revealed that the splicing inhibitory potency of the analogues and the modification of carboxylic acid moiety are well correlated with the antiproliferative activity against cancer cell lines [163,164]. 

The plant-derived splicing inhibitor, isoginkgetin, was identified from the leaves of the gingko tree by the screening of its mRNA splicing inhibitor [165]. Biflavones such as isoginkgetin belong to a subclass of the plant flavonoid family, which were reported to show anti-cancer activity [166]. Isoginkgetin inhibits splicing by preventing the stable recruitment of the U4/U5/U6 tri-small nuclear ribonucleoprotein, resulting in the accumulation of prespliceosomal A complex [165].

#### 4.1.2. Molecular Mechanism of Splicing Investigated by Splicing Inhibitors

Inhibitors introduced in this review were also used to provide detailed mechanistic insight into spliceosome formation and remodeling, as well as its impact on gene expression in cells. 

SF3B1 inhibitors are powerful tools to elucidate the function of SF3B1 and the SF3B complex in complicated splicing mechanisms such as the spliceosome assembly. Previously, the association of SF3B1 with the recruitment and/or stabilization of the U2 snRNP at the BP was reported [100,167,168,169,170]. However, the mechanisms of BP recognition by the U2 snRNP and SF3B1 are still unknown. The study, using the chemical compound, E7107, which affects U2 snRNP interactions at the BP, concluded that SF3B1 plays a role in mediating a conformational change of the U2 snRNP [154]. Similarly, SSA reduces the fidelity of U2 snRNA interactions with the BP, suggesting that SF3B1 participates in BP discrimination [153]. Cryo-EM structure analysis of the SF3B complex with or without E7107 showed that the compound bound to the mRNA-unbound SF3B complex and this SF3B complex was different from the RNA-bound closed conformation. This provided evidence that RNA and possibly other splicing factors trigger a conformational change in the SF3B complex for the spliceosome assembly, and E7107 causes splicing abnormalities by disrupting the conformational change [171]. In addition, using SSA and PlaB, it was reported that SF3B1 is involved not only in the early splicing reaction by BP recognition but also in the exon-ligation reaction [172], suggesting additional roles for SF3B1 throughout the splicing process.

The relationship between splicing factor and SUMOylation, a post-translational modification, was partially clarified by hinokiflavone, which is an analogue of isoginkgetin [173]. Previously, spliceosomal proteins were revealed as SUMO conjugation targets; however, little is known about the involvement of SUMO in spliceosome biogenesis and splicing regulation [174]. Hinokiflavone induced SUMOylation of pre-mRNA splicing factors, which contain six components of the U2 snRNP spliceosome subunit, by inhibiting SUMO protease SUMO-specific peptidase 1 (SENP1) activity. Consequently, hinokiflavone prevented transition of the spliceosome from its A to B complexes, resulting in global splicing modulation. It was also reported that inhibition of pre-mRNA processing factor 3 (PRPF3) SUMOylation prevented the interaction of U4/U6 di-snRNP with U5 to form tri-snRNP [175]. These reports suggested the notion that SUMOylation cycles were involved in spliceosome assembly and catalytic activity, and that they also affected alternative splicing regulation. An investigation using a compound like hinokiflavone is expected to facilitate the relationship between SUMO modification and alternative splicing.

These splicing-modulating compounds generally function as splicing inhibitors. However, affected transcripts are part of mRNAs. The sequence features which caused aberrant splicing by SSA and sudemycin, E7107, and H3B-8800 treatment were analyzed from recent transcriptome analyses [156,157,176]. Commonly, compound-induced retained introns are typically shorter and display a higher GC content and weaker polypyrimidine tracts and BP sequence. In addition, the presence of multiple BPs has an important role in determining sensitivity [156]. Interestingly, despite their structural similarities, SSA and sudemycin show common and differential effects on splicing regulation. SSA generally displays stronger effects on intron retention, and sudemycin affects exon skipping [156]. H3B-8800 is also more effective than E7107 on short introns that are rich in GC content [157]. Understanding of the relationships between these compounds and the differences in genome-wide splicing control will provide support for high-resolution observations of the splicing mechanism.

#### 4.1.3. Focusing on Splicing Regulation for Therapeutics of Cancer

Mutations of splicing factors found in various cancers are all heterozygous, probably because wild-type splicing factor has an essential role for cell survival in these cancers. Cancers that have the splicing factor mutation, therefore, show higher sensitivity to splicing inhibitors [177]. Some cancers without the splicing factor mutation also show sensitivity to splicing inhibitors. This observation is explained by the stress in splicing because primary transcripts are more abundantly expressed in cancer cells than in normal cells [178]. In fact, as previously mentioned, many splicing inhibitors were discovered to be cytotoxic compounds to cancer cells in early research. Based on this information, a splicing inhibitor is expected to act as an anti-cancer drug with a new mechanistic movement (Figure 3A).

The first splicing inhibitor to enter clinical trials was pladienolide derivative E7107 [179,180]. Unfortunately, this trial was discontinued because of vision loss occurring in some study participants [180]. Recently, H3B-8800 showed greater preferential cytotoxicity in spliceosome-mutant cells than E7107 by retaining short and GC-rich introns, which are enriched in genes encoding spliceosome components [157]. The enrichment of retained introns in mRNAs encoding spliceosome factors provides a rationale for H3B-8800 giving a preferential killing effect to spliceosome-mutant cancer cells compared with normal cells with a wild-type spliceosome. Consequently, H3B-8800 selectively killed acute myeloid leukemia cells and xenograft tumors. H3B-8800 entered phase 1 clinical trials [157], and it is expected to be the first anti-cancer drug with splicing inhibition (Figure 3B).

### 4.2. Compounds Regulating mRNA Splicing by G-Quadruplex Control

Initially, compounds with regulating rG4 structures associated with translational control were explored. RGB-1, RR82, and RR110, which bind selectively to rG4, affect the stability of NRAS mRNA rG4 and the translational efficiency of the NRAS 5′ UTR (Table 2) [181,182]. TRF2 is a protein with a central role in telomere maintenance. Three bisquinolinium compounds, 360A, PhenDC3, and PhenDC6, potentially bind to the TRF2 mRNA rG4 to alter its translation [183]. In addition, a compound that affected alternative mRNA splicing through regulating rG4 structures was also found. Previously, it was shown that there are two rG4 forming sites in Bcl-X mRNA, each being located close to an individual alternative 5′ ss. The compound, GQC-05, affects only one site by enhancing the stability of rG4 dependent on its structure [184]. The binding results in the reduced usage of the major 5′ ss that expresses the anti-apoptotic isoform of Bcl-X and the increased usage of an alternative 5′ ss that expresses a pro-apoptotic isoform. Moreover, alteration of splicing induces apoptosis. It was also reported that emetine and its analogue, cephaeline, disrupted rG4, resulting in the inhibition of rG4-dependent alternative splicing. Transcriptome analysis revealed that emetine globally regulates alternative splicing with variable exons that contain rG4 near proximal splice sites. This analysis revealed that rG4 controls alternative splicing at a genome-wide scale. Interestingly, emetine promotes the EMT state, suggesting that small molecules may alter cell fates associated with cancer progression [72].

Regulation of mRNA processing by rG4 is still unclear. Newly identified compounds that regulate rG4 will uncover the underlying RNA processing mechanism by rG4. In addition, several studies also indicated that rG4 structures are associated with human diseases, including neurological disorders [99] and cancer [185,186]. Uncovering these relationships will aid in therapy against diseases caused by rG4.

### 4.3. Food-Derived Natural Compounds Capable of Controlling mRNA Splicing

The above compounds are anticipated for clinical use. In addition, food-derived natural compounds with similar activity to the above compounds may function as anti-cancer drugs. Recent studies suggested that compounds originating from food also contain various anti-cancer activities, showing a variety of physiological activities within cells and the whole body (Figure 3B). For example, daily intake of bioactive food compounds is expected to be effective for preventing chronic diseases. Polyphenols and carotenoids have antioxidant activity and function to prevent lifestyle-related diseases [187,188,189]. In this section, we discuss some reports that were published on food-derived natural compounds with the ability to modulate mRNA processing.

Resveratrol is a polyphenolic flavonoid found in grape skins, grape seeds, and red wine [190], and it was shown to protect against cardiovascular disease, type 2 diabetes, and neurological disorders (Table 3) [190,191,192]. It was found that resveratrol modulates alternative splicing of SRSF3 and SMN2 mRNAs. The splicing abnormalities of these mRNAs are associated with the above-mentioned diseases [193]. This effect may be partly due to the ability of resveratrol to affect the protein level of several RNA processing factors, ASF/SF2, hnRNP A1, and human antigen R (HuR). Previously, although the SIRT1 protein was regarded as a major target of resveratrol, the knockdown of SIRT1 did not modulate alternative splicing of SRSF3 and SMN2 mRNAs, suggesting that another splicing regulatory protein is regulated by resveratrol for modulating these mRNAs.

Caffeine, abundant in coffee and tea, modulates the cell cycle and growth arrest, and induces apoptosis via the expression of various alternatively spliced p53 isoforms [194]. In one study, caffeine altered the expression levels of the p53α and p53β isoforms that are mediated by the downregulation of the SRSF3 mRNA and protein. In addition to p53 isoforms, other SRSF3 target genes were also alternatively spliced in response to caffeine treatment [195]. That study provided a new pathway of caffeine-modulated tumor suppression via the alternative splicing of target genes of SRSF3. 

Curcumin is a dietary polyphenolic compound enriched in the roots of turmeric with a broad therapeutic potential for cancer because of curcumin’s antitumor activity in various cancer cells [196,197]. Recently, it was reported that curcumin caused a splicing switch from a cancer-specific PKM2 isoform to normal PKM1 isoform in head and neck cancer cells, resulting in reduced tumor growth. In addition, global transcriptome analysis of curcumin-treated cells revealed curcumin’s effect on the alternative splicing of various genes involved in head and neck cancer [198]. 

We examined whether food-derived compounds provide inhibitory activity toward mRNA processing in the nucleus using a previously established screening system [199,200]. We identified the activity of inhibiting mRNA processing in the soybean-derived isoflavonoid fraction [200,201]. Furthermore, this activity was mainly exerted by compounds with a flavone skeleton [200]. Among them, apigenin and luteolin exhibited the strongest activity among 21 compounds with a flavone skeleton [202]. These compounds most intensely interacted with the U2 and U5 snRNP, suggesting that apigenin and luteolin were associated with spliceosomal components to directly prevent the function of spliceosomes, thus affecting alternative splicing at the genome-wide level. We observed more prominent sensitivity of tumorigenic cells to apigenin and luteolin than that observed for nontumorigenic cells, suggesting the potential anti-cancer activity of these compounds. We also screened for active constituents from spices and detected the inhibition of mRNA processing activity in ginger, cinnamon, and clove extracts. It seems that there may be many constituents with inhibitory activity toward mRNA processing in spices. Furthermore, 6-gingerol and 6-shogaol, active components in ginger, showed inhibitory activity toward mRNA processing [203]. The anti-cancer activity that was observed in ginger extract and 6-gingerol [204] might be partly exerted through anti-mRNA processing activity.

## Figures and Tables

**Table 1:** Chemical compounds which control splicing.

Name of Compounds and Chemical Formulas	Origin and Reference	Features of Inhibitor
pladienolide B (PlaB) 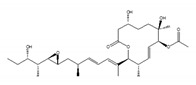	be derived from *Streptomyces platensis* Mer-11107 [149]	inhibit splicing
E7107 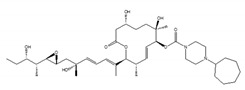	an analog of PlaB [149,150,151,152,153,154,155,156]	directly bind to SF3B complex and inhibit SF3B to interact with pre-mRNA
H3B-8800 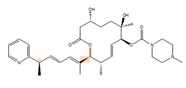	an analog of PlaB [157]	have entered clinical trials as anti-cancer drug because of preferential killing effect to spliceosome-mutant cancer
FR901464 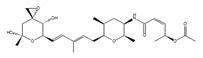	be derived from fermented broth of bacterium *Pseudomonas* sp. [158]	inhibit splicing
spliceostatin A (SSA) 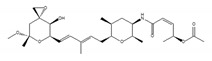	an analog of FR901464 [153]	directly bind to SF3B complex and inhibit SF3B to interact with pre-mRNA
sudemycin C1 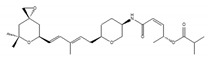	be designed based on pharmacophore model between FR901464 and PlaB [159,160]	exhibit better chemical stability than SSA and PlaB
GEX1A 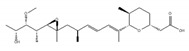	be derived from a culture broth of *Streptomyces* sp. [161,162]	directly bind to SF3B complex and inhibit SF3B to interact with pre-mRNA
9g, synthetic analogue of GEX1A 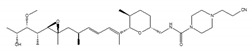	an analog of GEX1A [163,164]	be expected as the lead compound for the development of novel antitumor agents
isoginkgetin 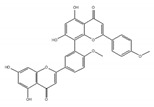	be derived from the leaves of the gingko tree [165,166]	prevent transition of the spliceosome
hinokiflavone 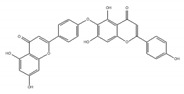	an analog of isoginkgetin [173]	induce SUMOylation of splicing factors by inhibiting SENP1 activity, and prevent transition of the spliceosome

**Table 2:** Modulators of G-quadroplex which control translation or splicing.

Name of Compounds and Chemical Formulas	Origin and Reference	Features of Inhibitor
RGB-1 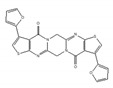	synthetic molecules [181,182]	modulate translational efficiency of TRF2
RR82 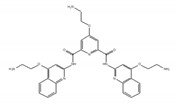	synthetic molecules [181,182]	modulate translational efficiency of TRF2
RR110 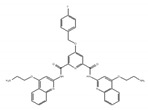	an analog of RR82 [181,182]	modulate translational efficiency of TRF2
360A 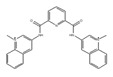	synthetic molecules [183]	modulate translational efficiency of TRF2
PhenDC3 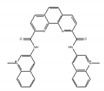	synthetic molecules [183]	modulate translational efficiency of TRF2
PhenDC6 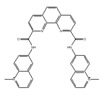	an analog of PhenDC3 [183]	modulate translational efficiency of TRF2
GCQ-05 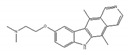	synthetic molecules [184]	modulate alternative splicing of Bcl-X
emetine 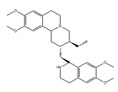	be contained in ipecas root [72]	modulate alternative splicing with variable exons which contain rG4 near proximal splice sites
cephaeline 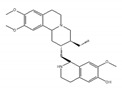	an analog of emetine [72]	show similar effect with emetine

**Table 3:** Food derived compounds which control mRNA processing or splicing.

Name of Compounds and Chemical Formulas	Origin and Reference	Features of Inhibitor
Resveratrol 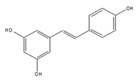	be contained in grape skins, grape seeds and red wine [191,192,193,194]	modulate alternative splicing of SRSF3 and SMN2
Caffeine 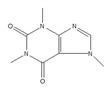	be contained in coffee and tea [195,196]	modulate alternative splicing of p53, and SRSF3 target mRNAs
Curcumin 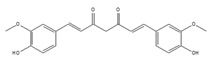	be contained in the roots of turmeric [197,198,199]	modulate alternative splicing of PKM2 and various mRNAs involved in head and neck cancer
apigenin 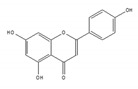	be contained in parsley and celery [203]	interact with the U2 and U5 snRNP and affect alternative splicing
luteolin 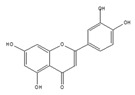	be contained in parsley and celery [203]	show similar effect with apigenin
6-gingerol 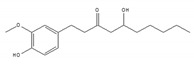	be contained in ginger [204]	inhibit mRNA processing
6-shogaol 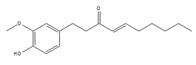	be contained in ginger [204]	inhibit mRNA processing
